# Sensing with Thermally Reduced Graphene Oxide under Repeated Large Multi-Directional Strain

**DOI:** 10.3390/s24175739

**Published:** 2024-09-04

**Authors:** Armin Yazdi, Li-Chih Tsai, Nathan P. Salowitz

**Affiliations:** 1 Department of Civil and Environmental Engineering, University of Wisconsin Milwaukee, Milwaukee, WI 53211, USA; 2 Department of Biomedical and Health Informatics, University of Wisconsin-Milwaukee, Milwaukee, WI 53211, USA; lctsai@uwm.edu; 3 Department of Mechanical Engineering, University of Wisconsin-Milwaukee, Milwaukee, WI 53211, USA

**Keywords:** sensors, strain, reduced graphene oxide

## Abstract

This paper presents a recent investigation into the electromechanical behavior of thermally reduced graphene oxide (rGO) as a strain sensor undergoing repeated large mechanical strains up to 20.72%, with electrical signal output measurement in multiple directions relative to the applied strain. Strain is one the most basic and most common stimuli sensed. rGO can be synthesized from abundant materials, can survive exposure to large strains (up to 20.72%), can be synthesized directly on structures with relative ease, and provides high sensitivity, with gauge factors up to 200 regularly reported. In this investigation, a suspension of graphene oxide flakes was deposited onto Polydimethylsiloxane (PDMS) substrates and thermally reduced to create macroscopic rGO-strain sensors. Electrical resistance parallel to the direction of applied tension ($\hat{x}$) demonstrated linear behavior (similar to the piezoresistive behavior of solid materials under strain) up to strains around 7.5%, beyond which nonlinear resistive behavior (similar to percolative electrical behavior) was observed. Cyclic tensile testing results suggested that some residual micro-cracks remained in place after relaxation from the first cycle of tensile loading. A linear fit across the range of strains investigated produced a gauge factor of 91.50(Ω/Ω)/(m/m), though it was observed that the behavior at high strains was clearly nonlinear. Hysteresis testing showed high consistency in the electromechanical response of the sensor between loading and unloading within cycles as well as increased consistency in the pattern of the response between different cycles starting from cycle 2.

## 1. Introduction

This paper presents recent investigations into the electrical-resistive properties of reduced graphene oxide (rGO) subjected to repeated strains up to 20.72%, with electrical measurements taken in multiple directions relative to the applied tensile strain for potential applications as a multi-directional strain sensor.

Strain and deformation represent some of the basic stimuli that can be *directly* sensed [1]. In many applications, strain sensors are incorporated into *hybrid* or *complex* sensors to measure other stimuli, with applications in aerospace engineering, robotics, structural health monitoring, and medical fields [2,3,4,5,6,7,8,9,10,11,12]. Wearable strain sensors are one of the most interesting applications of the strain sensors in the field [13,14], whose improvements and findings can be employed for medical applications [13].

Conventionally, the sensitivity of strain sensors is reported as *gauge factor* (GF), calculated according to Equation (1), where Δ*R* is the change in electrical resistance, R_0_ is the initial resistance (in an unstrained state), and *ε* is the strain [1]. Most strain sensors are unidirectional devices, requiring a rosette of 3 sensors to determine the state of plane strain.

(1) $$GF=\frac{\frac{\Delta R}{R_{0}}}{\varepsilon }$$

Electrical resistance can be calculated for solid materials using Equation (2), where *ρ*, *l*, and A are the resistivity, length, and cross-sectional area of the material, respectively.

(2) $$R=\rho \cdot \frac{l}{A}$$

Unlike resistivity in solid materials, resistance in some nano-particulate material structures, like graphene nanosheets or single-wall carbon nanotubes, can be dependent on the thickness of the film following the Power Law. Resistivity in these material structures can be calculated by Equation (3), where $t_{c}$ is the minimum thickness at which the particles form electrical paths, smaller than which no conduction occurs; $t_{min}$ is the thickness, above which the resistivity is not thickness-dependent; and n is the percolation exponent [15,16,17,18,19,20,21].

(3) $$\rho_{percolative}=\rho \cdot {\left(\frac{t_{min}-t_{c}}{t-t_{c}}\right)}^{n}$$

Conventional metal foil strain gauges have limited strain tolerance and relatively low sensitivity, with common GF around 2 and fail at strains up to 5% [22,23,24,25]. A recent interest in strain sensing for highly flexible robotics and wearable devices [8] has generated a need for strain sensors that can function beyond the limits of metal foil strain gauges (i.e., strain > 5% and with higher sensitivity) [26]. Recent studies have regularly reported nanoparticle-based strain gauges with GF up to 200 and survival of strains up to 50% [27,28,29]. Unfortunately, most nanoparticle-based strain sensors are expensive to produce and are composed of toxic materials such as silver, nickel, or chrome nanoparticles [30,31,32].

Recent studies have shown graphene can be used as a strain sensor [33,34] and tolerate large strains (around 20%) with high sensitivity (GF around 200). Being carbon, graphene itself is not toxic. However, large-scale production of graphene monolayers is expensive and associated with complex procedures [35,36,37,38].

rGO possesses many of the beneficial properties of graphene and can be mass produced using easy and inexpensive techniques making it a great candidate for strain sensors. Raw graphene oxide can be created by simple processing of graphite, such as graphite oxidation and ball milling process [39], or high-shearing of pre-oxidized graphite [40], which can then be deposited directly on a structure and chemically reduced to produce rGO [41,42]. There are several methods to deposit graphene oxide (GO) directly onto a surface, including drop casting, layer by layer deposition, inkjet printing, etc. [43,44,45]. Furthermore, there are several methods to reduce the GO to form rGO, falling into the broad categories of thermal, chemical, and electrochemical reduction methods [46,47,48,49].

While many studies have been performed on the basic relations between strain and resistance in rGO [50,51], electromechanical properties of rGO subjected to large strains have not been well-studied. This study explores rGO as a strain sensor exposed to uniaxial tension, resulting in strains up to 20.72% in the $\hat{x}$-direction. The resistive response of the rGO was measured in multiple directions relative to the applied tension to identify their transfer functions.

## 2. Materials and Methods

Samples were created using drop-casting and thermal reduction techniques to deposit sensors on a highly stretchable substrate. Uniaxial tension was applied to the samples to generate strains up to 20.72%, and the electrical responses were measured. Further inspection of the samples and structures was performed using a confocal microscope.

### 2.1. Sample Synthesis

Samples were composed of a highly stretchable polymer substrate on which the GO was deposited, then reduced, and then electrical lead wires were attached. This study employed the synthesis method used by M. Rezaee et al. (2019) [41,45,52] and L. Tsai et al. (2019) [42,46,49].

To create the substrate, DOW SYLGARD 186 Polydimethylsiloxane (PDMS) [53] was cast into polycarbonate molds with length ($\hat{x}$), width ($\hat{y}$), and thickness ($\hat{z}$) of 127 mm, 15.24 mm, and 2.54 mm, respectively. A small reservoir was designed at the center of the molds with dimensions of 6.35 mm by 6.35 mm to confine the GO suspension when it was deposited. The PDMS was cured at room temperature for 3 days. The resulting PDMS substrate was washed with ultrapure deionized water from a milli-Q advantage A10 system [54] and was dried using compressed air.

To promote adhesion between the components of the test specimens produced, namely rGO and PDMS substrate, the PDMS was treated with oxygen plasma for 5 min using a PE-25 Plasma Etcher system [55] and then was immersed in a solution of 2% (3-Aminopropyl) triethoxysilane (APTES) to Ethanol for 3 h [42].

A 0.4 wt % dispersion of Graphenea GO, with graphene oxide flakes measuring roughly 5 μm wide by 0.3 nm thick, was agitated in an 80-watt CREWORKS Ultrasonic Cleaner for 2 min and then drop-cast onto the 6.35 mm by 6.35 mm reservoir using a pipette, producing a GO area density of 0.0069 $\frac{mg}{mm2}$ [56]. The GO was allowed to dry in ambient room conditions for 23 h. Specimens were placed in an OTF-1200 Series Split Tube Furnace(MTI Corporation, Richmond, CA 94804 USA) [57] under an Argon environment to be thermally reduced with temperature increasing from ambient to 180 °C in 60 min, maintained at a constant to 180 °C for 60 min, increased to 200 °C in 5 min, maintained at 200 °C for 10 min, and finally returned to room temperature in 90 min.

**Figure 1:**
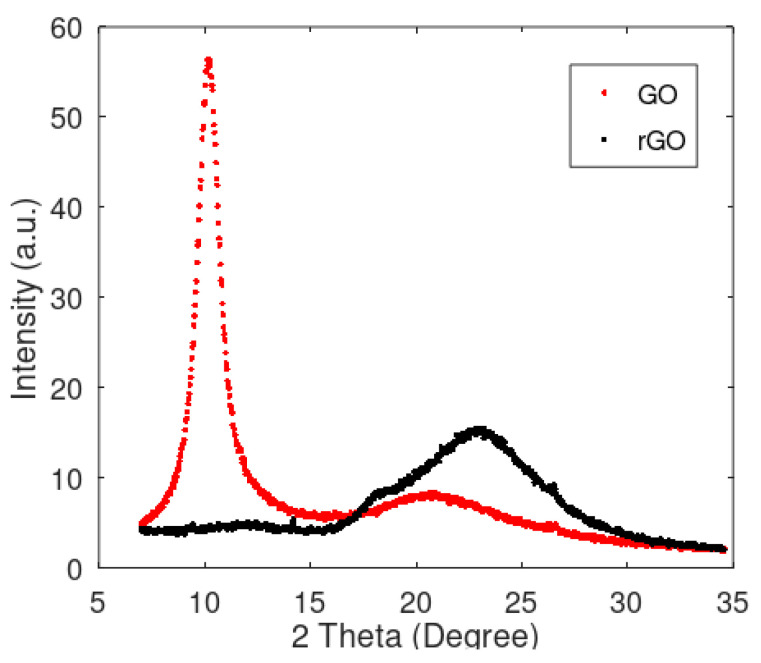
XRD analysis performed on GO and rGO.

Prior to the reduction process, the GO patch was functionally an insulator, with no or negligible measurable conductivity. After the reduction process, the patch demonstrated finite conductivity, with measurable resistance making it suitable for use as a resistive strain gauge. This change in behavior was attributed to the reduction in the oxygen-containing functional groups [58]. Analysis using X-Ray diffraction (XRD) was performed with a Bruker D8 Discover on samples before and after the reduction process to confirm the production of rGO. As shown in Figure 1, the first peak for the GO appeared at about 10.19°, which can be attributed to the interlayer spacing of about 0.87 nm. Thermal procession caused this peak to disappear, forming a new peak at 22.81°, which corresponded with the interlayer spacing of about 0.39 nm. This decrease in the interlayer spacing can be attributed to the oxygen functional group reduction in GO and is reasonably consistent with the literature indicating a reduction in GO [59,60,61,62]. 

**Figure 2:**
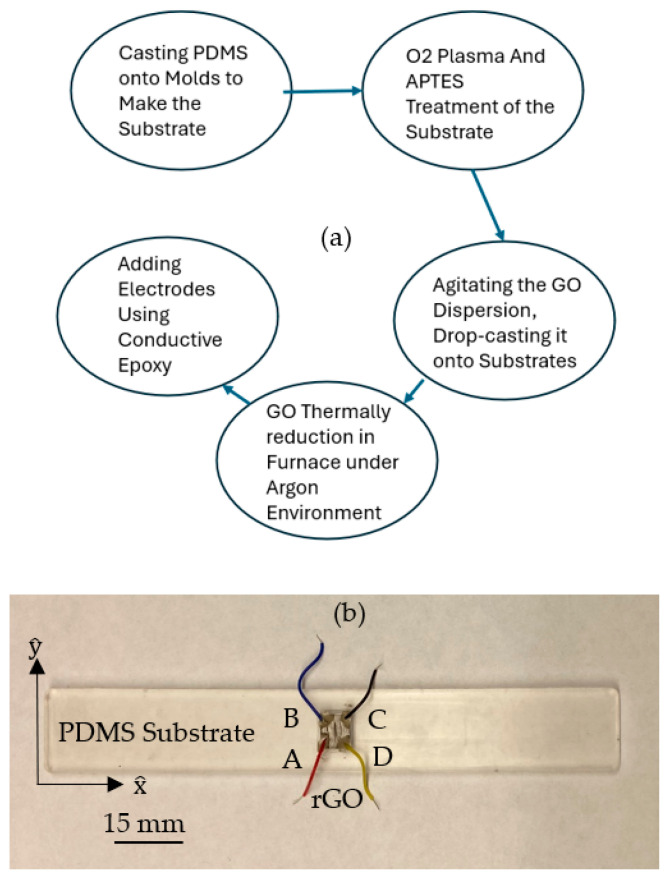
(**a**) Preparation of the sample and (**b**) tensile-testing samples.

The general synthesis procedure of the sample is shown in Figure 2a. As shown in Figure 2b, four electrodes and lead wires were applied to the rGO patch using CircuitWorks 2400 (CHEMTRONICS, Kennesaw, GA 30152, USA) conductive epoxy [63,64] at points A, B, C, and D. It is worth noting that orders of magnitude difference between resistance of electrodes wire and rGO made the piezo-resistivity of the wire negligible.

### 2.2. Imaging Using Confocal Microscope

High resolution images of the samples under strain were acquired with an OLYMPUS OLS4100 3D laser microscope (Olympus Corporation, Tokyo, Japan). Images of the rGO were taken while held at strains of 0%, 1.51%, 4.54%, 7.57%, 10.59%, 13.62%, 16.65%, and 19.68%.

### 2.3. Tensile Test Set-Up and Measurement Method

An INSTRON 5980 Series Universal Testing System was used to control the strain applied to the samples. 

To better constrain the ends of substrates for testing, load-plates were 3D printed using polylactide material measuring 15.24 mm by 15.24 mm by 2.54 mm. The ends of the PDMS substrates were each sandwiched between and adhered to two plates using Rhino cyanoacrylate adhesive. The distance between grips holding the samples initially was 96.52 mm [49].

**Figure 3:**
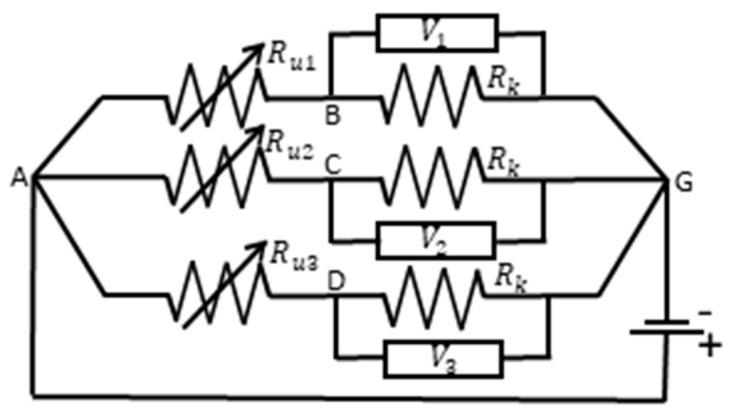
Data acquisition circuit diagram.

As shown in Figure 3, 5.6 kΩ resisters (R_k_) were connected in series with each resistive path, creating bridge circuits.

A 5-volt bias voltage was applied across the circuits, with electrode A as the ‘hot’ source, and the voltages were measured across each known resistor (V_i_) using a Tektronix 3014 oscilloscope. The measured voltages across known resistances allowed for calculation of the unknown resistances (R_ui_) using Equation (4), where the subscript i indicates the path of inspection (e.g., AB, AC, AD) [49]. Measured voltages less than or equal to 15 mV were considered as electrical failure.

(4) $$R_{ui}=\left(\frac{5}{V_{i}}-1\right)\cdot R_{k}$$

In total, 19 samples were created to test the resistive response of the rGO in 3 directions ($\hat{x}$, $\hat{y}$, and at a 45° angle to the direction of applied tension). The distance between electrodes AB, AC, and AD on average were measured in an unloaded state as 1.827 mm, 3.728 mm, and 1.541 mm with the standard deviations of 0.555 mm, 0.652 mm, and 0.529 mm, respectively. The deviations in distances were due to consistency in the application of a conductive epoxy to attach the electrodes and wires. Tensile displacements were applied in the $\hat{x}$-direction in 1 mm increments at a rate of 0.1 mm/sec and resistances measured up to a maximum of 20.72% strain.

### 2.4. Repeatability and Hysteresis

To better investigate the *repeatability* of the samples, samples 4, 9, 12, and 18 were randomly selected to be tested under cyclic tensile loading. The results are presented in Section 3.5. The experiment was conducted at room temperature and at almost consistent humidity.

Moreover, to investigate the hysteresis behavior, a new sample with 0.61 mm distance between electrodes was tested, undergoing 20 cycles of tensile loading and unloading in the range of 0% and 20.72% strains with the pace of 0.2 mm/s. Data were collected at each 1 mm interval. Results are shown in Section 3.6.

## 3. Results and Discussion

### 3.1. Microscopic Imaging

#### 3.1.1. Propagation and Accumulation of Cracks

**Figure 4:**
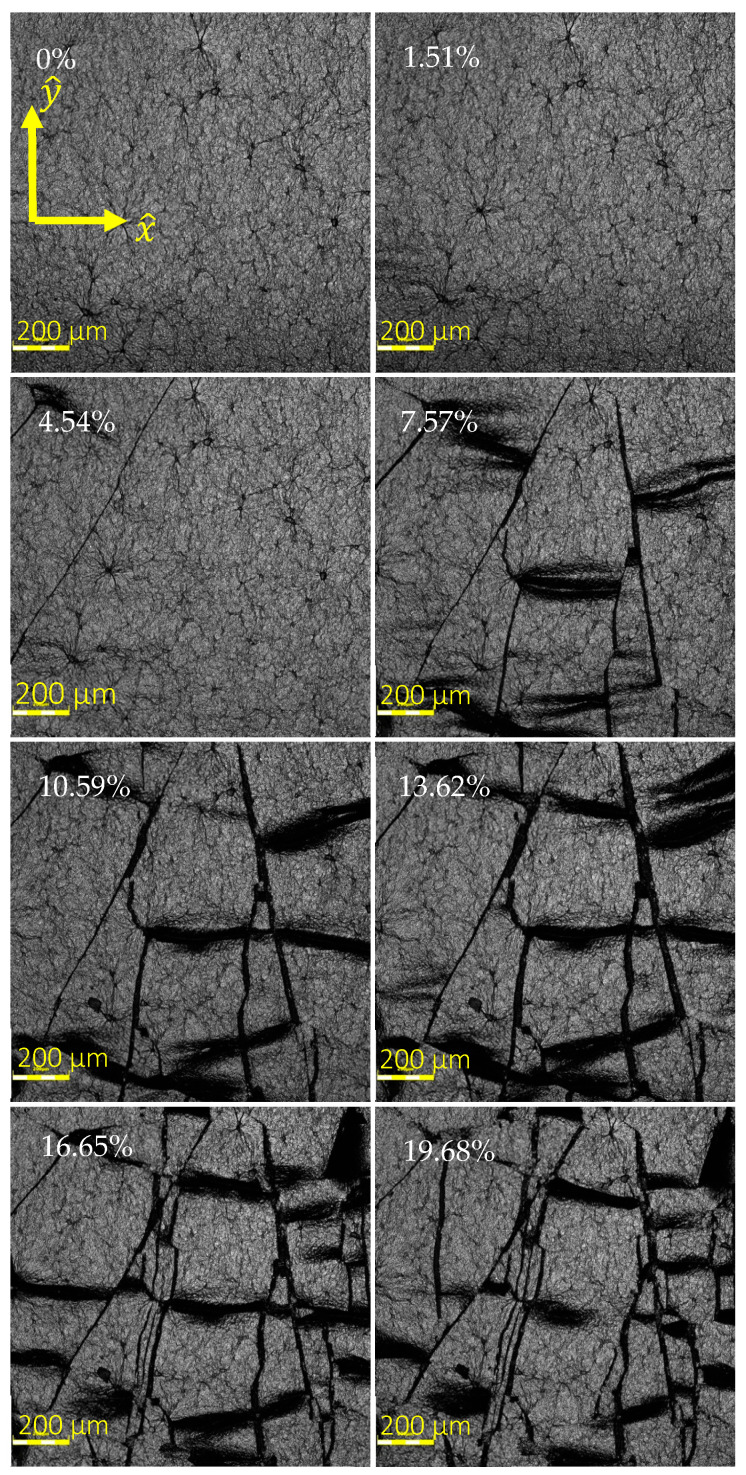
Propagation of the cracks and sliding of the rGO flakes over each other.

Microscopic inspection, shown in Figure 4, indicates that there was no significant brand-new cracking observed in the rGO at strains less than 7.57%. As can be seen, significant cracking and displacements accumulated at a faster rate, with strains beyond 7.25%. The approximate accumulated length of the visible cracks at 0%, 1.51%, and 4.54% can be manually measured using the scale bar as 0, 0, and 1.64 mm, respectively, whereas at strain of 7.57%, this value increased drastically to the value of 7.62 mm and then increased to 8.74 mm at 10.59% strain. This observation matches the physical mechanism underlying nonlinear percolative-like behavior of the material discussed in Section 3.2. The variation in the strain value at which the nonlinear trend begins can be attributed to the cracks that may occur at the locations of electron paths between electrodes before or after the 7.25% strain is reached. 

#### 3.1.2. Locally Distributed Cracks

Furthermore, the same images suggest that the cracks aligned with $\hat{x}$-direction, disconnecting electrical paths in $\hat{y}$-direction, were only locally distributed rather than completely across the film and were relatively small in length causing relatively smaller resistance changes, which justifies the relatively small magnitude of $\frac{\Delta R}{R_{0}}$ in the $\hat{y}$-direction discussed in Section 3.3. 

#### 3.1.3. Crack Propagation and Stabilization after the First Cycle

**Figure 5:**
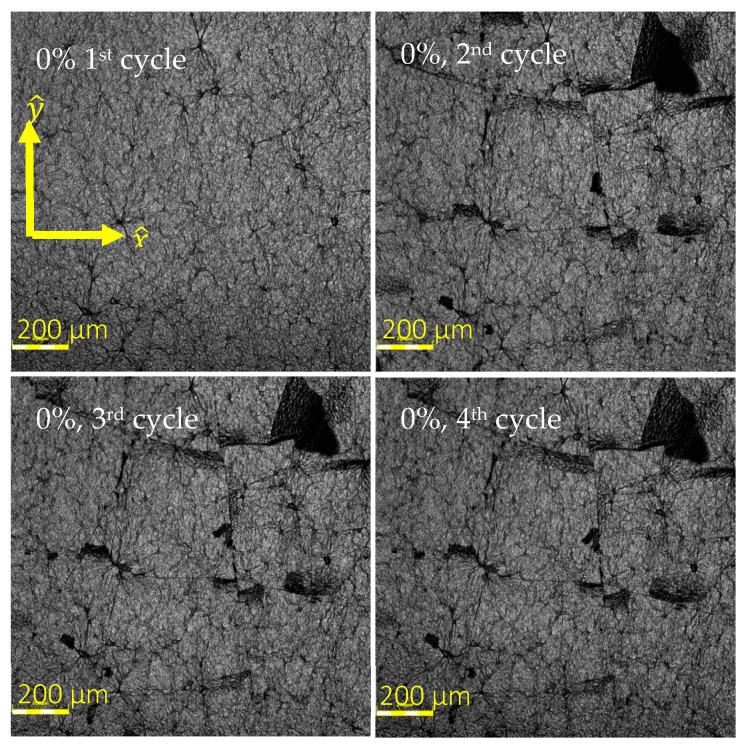
Permanent microcracks occurring after the first cycle of tensile loading.

By application of 4 repeated cyclic loads, during the first cycle of strain, formation of cracks was observed in the rGO, explaining the drastic change in behavior. Upon releasing the samples from the first cycle of strain, the cracks tended to close, and the shifted rGO flakes returned to their original location. However, very small gaps between the edges of former cracks remained in place in the size of micro-cracks (Figure 5). Upon subsequent cycling, these residual micro-cracks would open and close, demonstrating a more consistent mechanical behavior, which explains the large shift in RSD between cycle 1 and 2 and stabilization of the RSD value starting from cycle 2 discussed in Section 3.5.

### 3.2. Relative Resistance Change in the $\hat{x}$-Direction (between A and D)

#### 3.2.1. Experimental Observation

**Figure 6:**
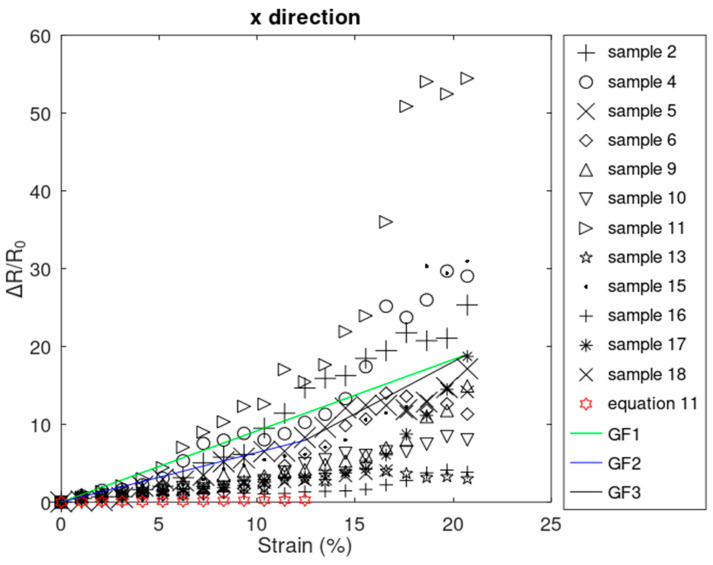
Linear–nonlinear resistive response in the $\hat{x}$-direction in samples that survived throughout the experiment.

The electromechanical behavior of the rGO films under tension generally fell into two regions, as can be seen in Figure 6. In the first region, relative resistance change ($\frac{\Delta R}{R_{0}}$) is increasing and highly linearly proportional with the strains between 0% and a certain strain, which can range from about 3% to 12% in the $\hat{x}$-direction. 

In this region, rGO’s resistive response is similar to that of solid materials, described as piezoresistive behavior. Beyond this strain (region 2), the rGO film’s relative resistance change is increasing as well but with a nonlinear trend, like particle-based sensors which are modeled as percolative.

#### 3.2.2. Mathematical Analysis

- Region 1

Application of uniaxial tension to specimens in the $\hat{x}$-direction resulted in a change in length (l) in the $\hat{x}$-direction and, consequently, the strain in the $\hat{x}$-direction (ε). This also produced changes in the perpendicular width (w) in the $\hat{y}$-direction and strain through Poisson effects. Discussion of thickness (t) refers to the $\hat{z}$ direction (mutually perpendicular to $\hat{x}$ and $\hat{y}$ following a right-handed convention). Original lengths (l, w, t) without applied strain are noted with the ${\mathrm{subscript}}_{\;0}$, and lengths resulting in the applied strain in the $\hat{x}$-direction are noted with the ${\mathrm{subscript}}_{\;\varepsilon }$. Because of the relative thickness of the rGO compared to the PDMS, the PDMS structure was assumed to dominate and dictate deformation of the rGO patch in plane. Specifically, the Poisson effect in the PDMS would govern the change in width ($\hat{y}$ dimension) of the rGO. These deformations can be calculated by Equations (5) and (6), respectively [15,17,18,19,20], where $l_{0}$ and $w_{0}$ are the original length and width of the rGO film, and ν is the Poisson’s ratio of the PDMS, which ranges from 0.45 to 0.5 [65]:

(5) $$l_{\varepsilon }=l_{0}(1+\varepsilon )$$

(6) $$w_{\varepsilon }=w_{0}(1-\nu \;)$$

The average thickness of the rGO patch can be calculated as Equation (7), where $t_{0}$ is the original thickness of the rGO film, and ν is the Poisson’s ratio of the PDMS [15,17,18,19,20]:

(7) $$t_{\varepsilon }=\frac{t_{0}}{\left(1+\varepsilon \right)\left(1-\nu \;\varepsilon \right)}$$

The resistance of the rGO under strain ($R_{\varepsilon }$) can be calculated according to Equation (8), where ρ is the resistivity of the rGO [1,66]:

(8) $$R_{\varepsilon }=\rho \cdot \frac{l_{\varepsilon }}{w_{\varepsilon }\cdot t_{\varepsilon }}$$

Using Equations (5)–(8), the resistance of the rGO patch in the unstrained state ($R_{0}$) and under strain ($R_{\varepsilon }$) can be calculated as Equations (9) and (10), respectively, where zero as the subscript denotes the original state of the parameter (i.e., $l_{0}$, $w_{0}$ and $t_{0}$):

(9) $$R_{0}=\rho \cdot \frac{l_{0}}{w_{0}t_{0}}$$

(10) $$R_{\varepsilon }=\rho \cdot \frac{l_{0}\cdot \left(1+\varepsilon \right)}{w_{0}\cdot \left(1-\nu \;\varepsilon \right)\cdot \frac{t_{0}}{\left(1+\varepsilon \right)\left(1-\nu \;\varepsilon \right)}}$$

Using Equations (9) and (10), relative resistance change can be calculated by Equation (11):

(11) $$\frac{\Delta R}{R_{0}}=\frac{R_{\varepsilon }-R_{0}}{R_{0}}=2\varepsilon +\varepsilon^{2}$$

Equation (11) shows a second-order polynomial, but because strains are small in this range, the strain squared term is very small, resulting in reasonably linear behavior (Figure 6) and an increasing trend, with a coefficient of determination of 99.98% and 100% for induced strains between 0 and 12%. This reasonably linear trend is consistent with the experimental results shown in Figure 6.

- Region 2

In region 2, nonlinear relations between strain in the $\hat{x}$-direction and relative resistance change were observed. This behavior is commonly observed in particulate materials and described by percolation theory. Assuming the rGO’s electromechanical properties in this region are thickness dependent and follow the Power Law, its resistivity can be calculated by Equation (3) [15,17,18,19,20,21].

Using Equations (2), (3), (5)–(7), the $\frac{\Delta R}{R}$ of the rGO can be calculated as Equation (12):

(12) $$\frac{\Delta R}{R_{0}}={\left(1+\varepsilon \right)}^{2}\cdot {\left(\frac{t_{0}-t_{c}}{t_{\varepsilon }-t_{c}}\right)}^{n}-1$$

The nonlinearity shown in the second region of Figure 6 can be attributed to the exponent 2 and n of (1+ε) and $\left(\frac{t_{0}-t_{c}}{t_{\varepsilon }-t_{c}}\right)$, respectively, as shown in Equation (12). The strain with the exponent of 2 was not neglected in this equation. Its inclusion in this Equation can enhance the precision of this Equation in this region, where strain and its squared values can be as high as 0.272 and 0.0739, respectively.

In this equation, t_c_, t_0_, and n are constant and positive values [18]. Taking the derivative of Equation (7) with respect to ε produces Equation (13):

(13) $$\frac{{dt}_{\varepsilon }}{d\varepsilon }=-t_{0}\frac{1-2\nu \cdot \varepsilon -\nu }{{\left(1+\varepsilon \right)}^{2}{\left(1-\nu \cdot \varepsilon \right)}^{2}}$$

Additionally, it is known that the Poisson’s ratio of the PDMS is less than 0.5, and the maximum strain applied is less than 20.72%. Therefore, the numerator of the fraction in Equation (13) is a positive value, and $\frac{{dt}_{\varepsilon }}{d\varepsilon }$, which is the slope of the $t_{\varepsilon }$ as a function of ε, is a negative value. This means that the average thickness of the film decreases with an increase in the applied tension. Therefore, with the sample being tensioned, with increase in strain and decrease in the average thickness [15,18,20,67,68], the relative resistance change increases according in Equation (12), which matches the increasing trend observed in the second region of Figure 6. 

These linear and nonlinear percolative-like behaviors match the significant cracking and displacements accumulated at a faster rate, with strains beyond 7.25%, which was discussed in Section 3.1.1.

The gauge factor was calculated with a linear fit approximation across the entire range from 0% to 20.72% and also piecewise linear fit with sections spanning 0% to 12.43% and 12.43% to 20.72% to support comparison to common foil strain gauges (with gauge factors around 2), even though region 2 clearly deviates from the linear trend. The linear gauge factors were found to be 91.50 $\frac{\Omega /\Omega }{m/m}$, 63.73 $\frac{\Omega /\Omega }{m/m}$, and 133.16 $\frac{\Omega /\Omega }{m/m}$, shown in Figure 6 as GF1, GF2, and GF3, respectively, which were significantly greater than that of common foil strain gauges (linear gauge factor about 2).

**Figure 7:**
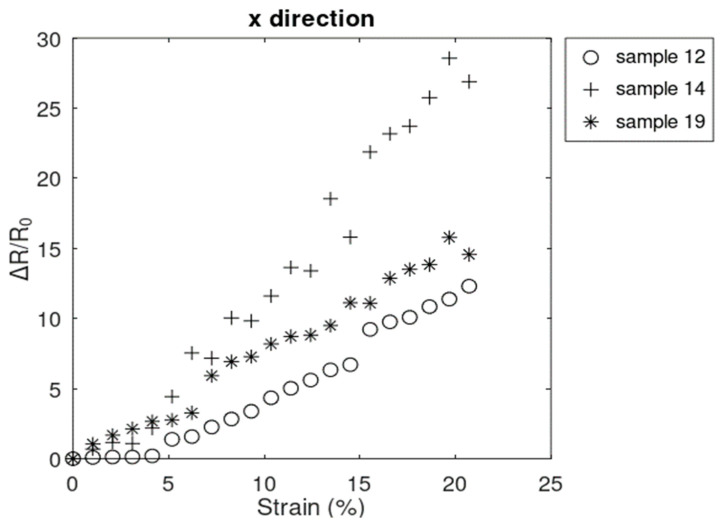
Only linear resistive response in the $\hat{x}$-direction.

Out of 15 samples that survived the strains up to 20.72%, 3 samples showed linear resistive behavior in the $\hat{x}$-direction throughout the experiment and did not enter the nonlinear region (Figure 7). Our initial investigations showed that in some cases, nonlinearity was initiated at higher strains. 

**Figure 8:**
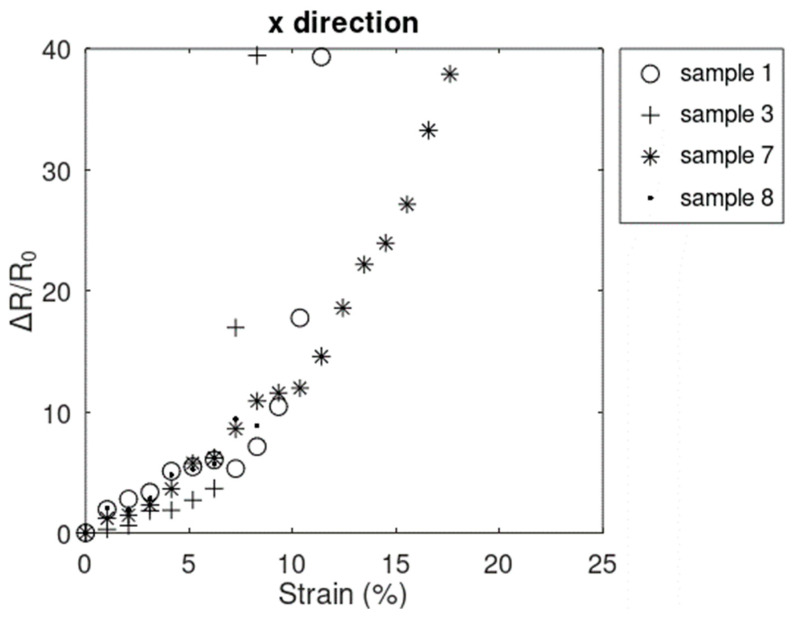
Samples that failed before 20.72% strain in the $\hat{x}$-direction.

As is shown in Figure 8, four samples failed prior to reaching the strain of 20.72%. All 4 samples showed nonlinear behaviors prior to failure.

### 3.3. Relative Resistance Change in the $\hat{y}$-Direction (between A and B)

**Figure 9:**
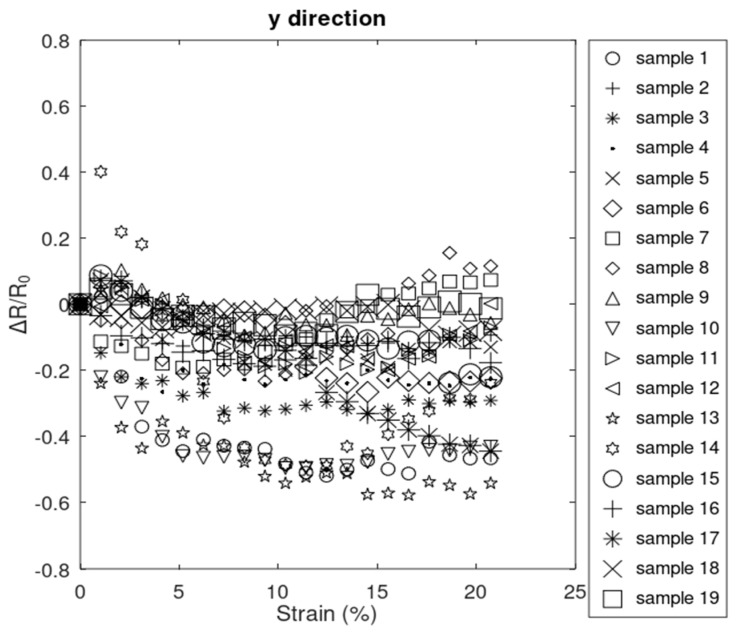
Relative resistance changes in the $\hat{y}$-direction.

The change in resistance measured in the $\hat{y}$-direction (perpendicular to the direction of applied tensile strain) revealed a generally decreasing trend, as shown in Figure 9, with some local fluctuations. These locally increasing trends in the relative resistance change can be attributed to the formation of locally formed cracks aligned with $\hat{x}$-direction, partially severing the interconnections between flakes in the $\hat{y}$-direction, as discussed in Section 3.1.2.

In the $\hat{y}$-direction, resistance can be calculated as Equation (14):

(14) $$R=\rho \cdot \frac{w}{l\cdot t}$$

Substituting Equations (5)–(7) into Equation (14), resistance ($R_{y}$) in the $\hat{y}$-direction can be obtained by Equation (15):

(15) $$R_{y}=\rho \cdot \frac{w_{0}{\left(1-\nu \cdot \varepsilon \right)}^{2}}{l_{0}\cdot t_{0}}$$

Relative resistance changes in the $\hat{y}$-direction ($\frac{\Delta R}{R_{y_{0}}}$) can be obtained using Equation (16), where $R_{y_{0}}$ is original resistance in the $\hat{y}$-direction and can be obtained by setting ε to zero in Equation (16):

(16) $$\frac{\Delta R}{R_{y_{0}}}=\frac{R_{y}-R_{y_{0}}}{R_{y_{0}}}=\nu^{2}\cdot \varepsilon^{2}-2\nu \cdot \varepsilon$$

Taking derivative of this equation with respect to ε, the slope of the $\frac{\Delta R}{R_{y_{0}}}$ can be obtained by Equation (17), which is a negative value, considering the range of Poisson’s ratio for PDMS (between 0.45 and 0.5) and the range of strain (<0.2072), which matches the general decreasing trend shown in Figure 9.

(17) $$\frac{\partial \frac{\Delta R}{R_{y_{0}}}}{\partial \varepsilon }={2\nu }^{2}\cdot \varepsilon -2\nu$$

Equations (16) and (17) were obtained assuming the bulk-like behavior of the rGO in the first region and that the resistivity was not a function of thickness. Assuming the percolative behavior of the rGO and that the resistivity is a function of thickness, the relative resistance change in the $\hat{y}$-direction can be obtained by Equation (18):

(18) $$\frac{\Delta R}{R_{y_{0}}}={\left(1-\nu \cdot \varepsilon \right)}^{2}\cdot {\left(\frac{t_{0}-t_{c}}{t_{\varepsilon }-t_{c}}\right)}^{n}-1$$

The magnitude of the responses in the $\hat{y}$-direction were significantly lower than that of $\hat{x}$-direction. This can be explained by the fact that the cracks largely propagated in the $\hat{y}$-direction and interrupted electron paths in the $\hat{x}$-direction. 

### 3.4. Relative Resistance Change at a 45° Angle (between A and C)

**Figure 10:**
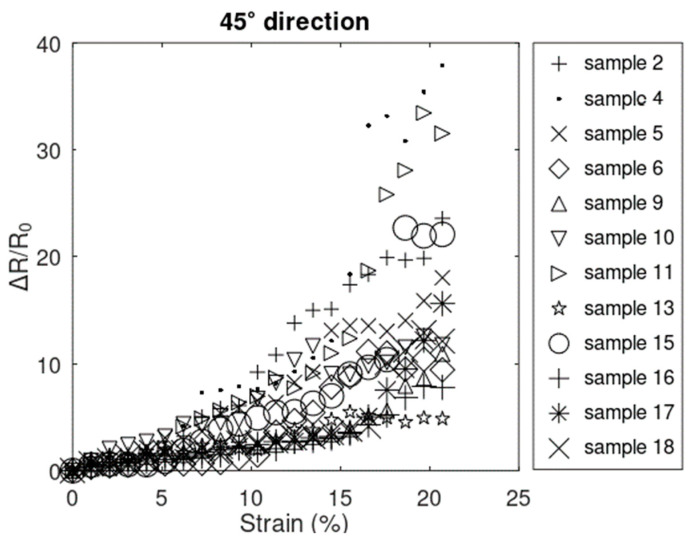
Resistive response of the samples with linear–nonlinear behavior in the 45° direction.

**Figure 11:**
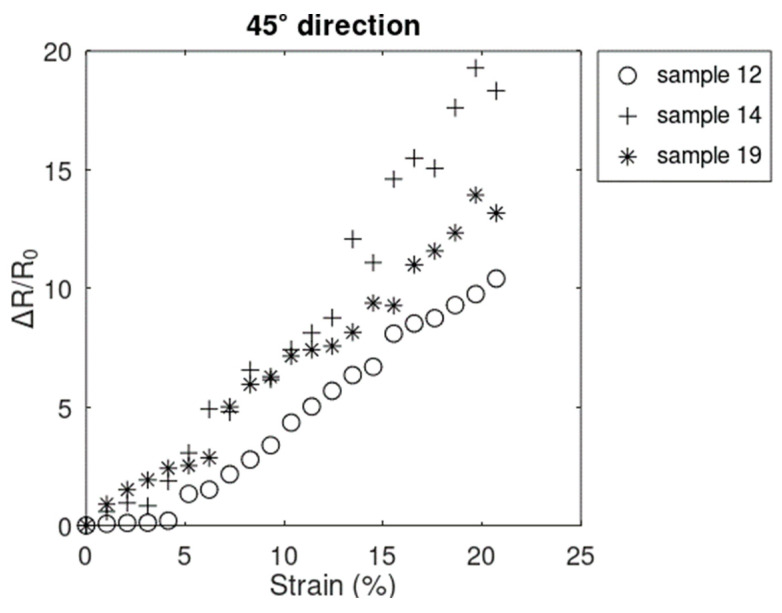
Resistive response of the samples with linear behavior in the 45° direction.

**Figure 12:**
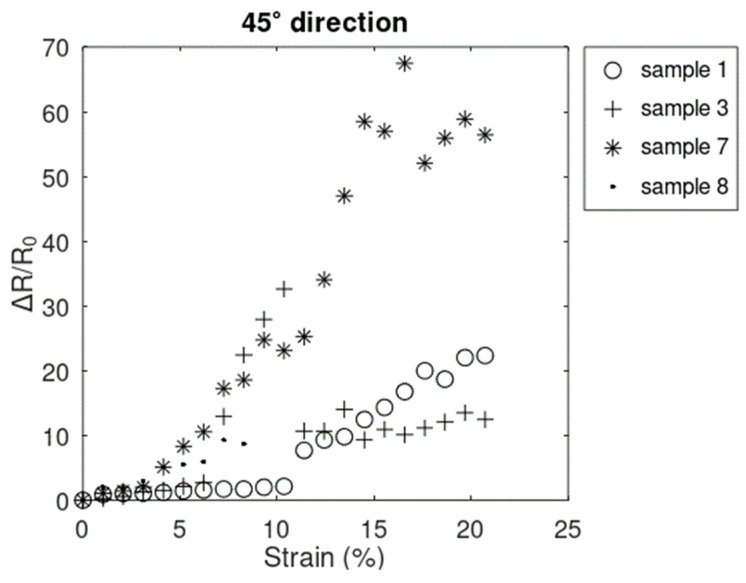
Resistive response in the 45° direction for the 4 samples that electrically failed in the $\hat{x}$-direction first.

Data collected between points A and C from 15 samples that did not fail before 20.72% showed the same general resistive behavior as in the $\hat{x}$-direction. The same 12 samples (Figure 10) and 3 samples (Figure 11) showed increasing linear–nonlinear and increasing only-linear behavior, respectively. Looking at the trend in the plot in both groups of samples, the resistive response most closely followed the trends in the $\hat{x}$-direction rather than the $\hat{y}$-direction. The smaller magnitude of the resistive response in the 45° direction, with respect to that of $\hat{x}$-direction, can be explained by the positive values of resistive response in the $\hat{x}$-direction being offset by the negative values in the $\hat{y}$-direction. It can also explain the reason why samples generally electrically survived longer in the *45°* direction (Figure 12) compared to the $\hat{x}$-direction (Figure 8). 

Strain transformation, to map a state of strain in a plane to rotated directions, is achieved using Equation (19) [69,70,71,72], where *θ* is the angle of rotation, $\varepsilon_{x}$ and $\varepsilon_{y}$ are normal components of the strains in the $\hat{x}$- and $\hat{y}$-directions, and $\gamma_{xy}$ is the shear component of the strain all in the original element.

(19) $$\varepsilon_{\theta }=\varepsilon_{x}\;{cos}^{2}\theta +\varepsilon_{y}{sin}^{2}\theta +\gamma_{xy}\cdot \;sin\theta \cdot cos\theta$$

Since samples were under uniaxial tension, γ_xy equals zero, and since the path between electrodes A and C was at a 45° angle to $\hat{x}$, the normal strain on the path from A to C can be calculated using Equation (19), which simplifies to Equation (20):

(20) $$\varepsilon_{45{}^{\circ}}=\frac{\varepsilon_{x}+\varepsilon_{y}\;\;}{2}$$

This equation clearly shows that the strain sensed between electrodes A and C was affected by the sum of the strains in the $\hat{x}$-and $\hat{y}$-directions, all divided by 2. While analysis of the strain–resistance relations in the $\hat{x}$- and $\hat{y}$-directions demonstrated differing responses, the result of Equation (19) suggests that the response at a 45° angle between them should be a weighted sum of the two. Since the magnitude of effects in the $\hat{y}$-direction was significantly smaller than that in the $\hat{x}$-direction, the resistive response of the sensor at the angle should be dominated by the resistive response in the $\hat{x}$-direction. 

### 3.5. Stabilization beyond Initial Strain Application and Removal

**Figure 13:**
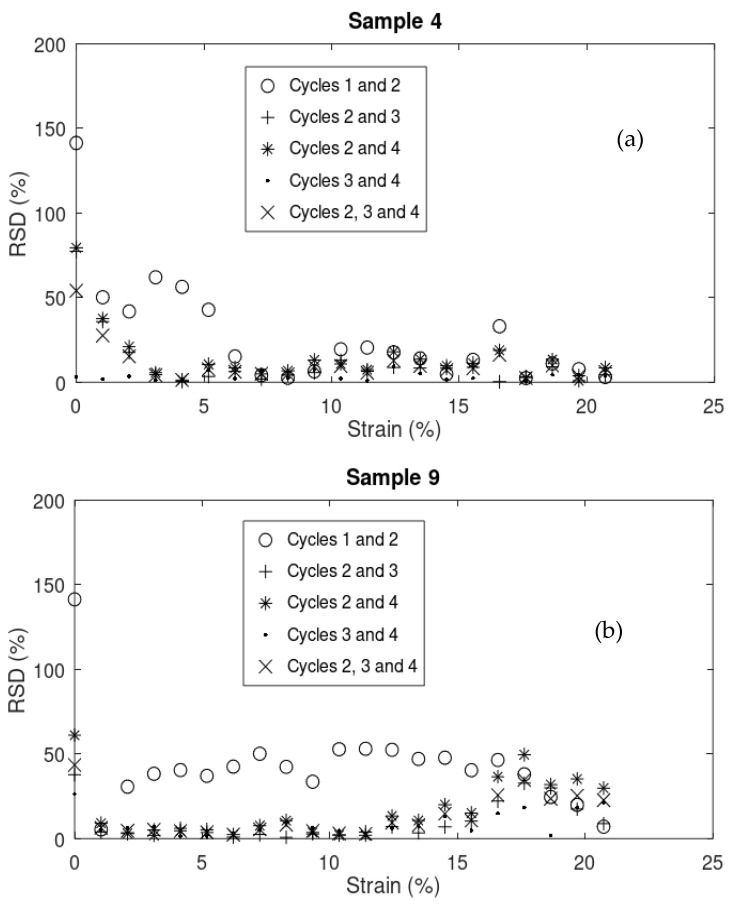
RSD of the data ($\frac{\Delta R}{R_{0}}$) between cycles in samples (**a**) 4 and (**b**) 9.

**Figure 14:**
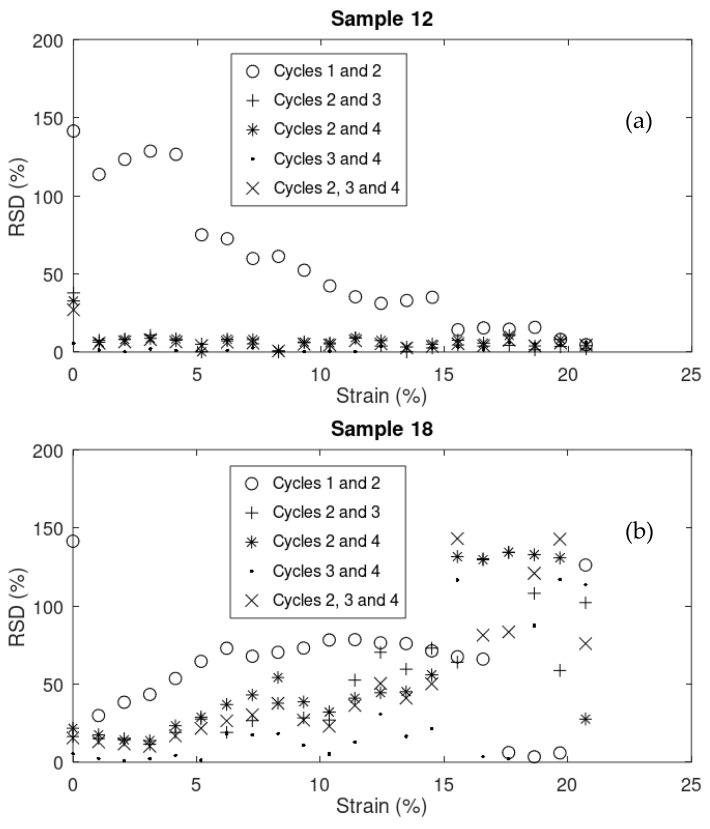
RSD of the data ($\frac{\Delta R}{R_{0}}$) between cycles in samples (**a**) 12 and (**b**) 18.

Figure 13a,b and Figure 14a,b compare the relative standard deviation (RSD) of the data ($\frac{\Delta R}{R_{0}}$) between cycles in 4 samples (4, 9, 12, and 18, respectively). The greater the RSD means, the higher the variation in the data between cycles. As it is shown in most cases, the RSD between cycles 1 and 2 is significantly higher than that between cycles 2 and 3, 2 and 4, 3 and 4, and even 2, 3, and 4 combined. This stabilization of electrical response can be attributed to the residual micro-cracks shown in Figure 5 and discussed in Section 3.1.3, which contribute to more consistent mechanical behavior starting from cycle 2.

### 3.6. Hysteresis and Recovery Analysis

**Figure 15:**
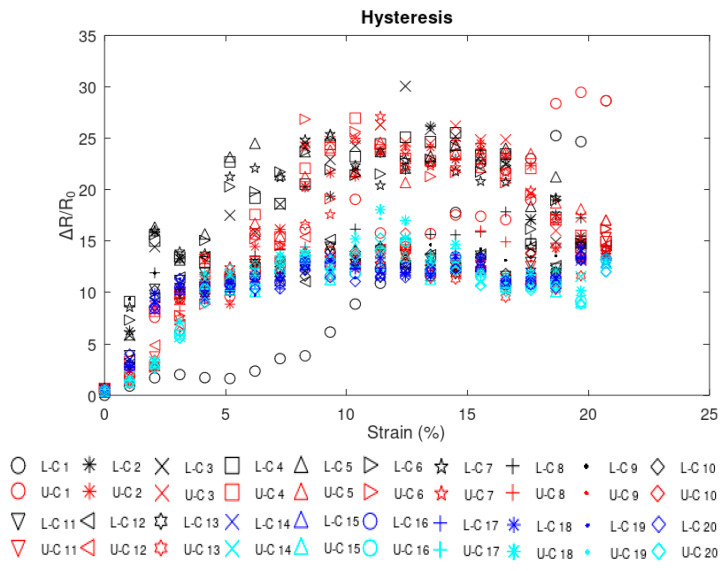
Hysteresis analysis.

An analysis of the differences in response through multiple loading and unloading cycles was also performed. The hysteresis analysis of the rGO sample is shown in Figure 15, where L-Ci and U-Ci stand for loading cycle number i and unloading cycle number i, respectively. It can be seen from the plot that the shift in the electromechanical response of the sensor between loading and unloading within cycles is small. Additionally, increased consistency in the pattern of the response between different cycles starting from cycle 2 can be observed. The largest change in behavior was observed over the first strain cycle, with an average of 5.6234 Ω/Ω difference between the loading and unloading data points. This initial cycle stabilized behavior in following cycles. 

In another study conducted by A. Yazdi (2023) [73], samples with the same GO area density of 0.0069 $\frac{mg}{mm2}$ were held at 7.57% strain for up to 40 days (57,436 min). At most, the sensor showed an about 22 $\frac{\Omega }{\Omega }$ shift in relative resistive change. After being released, it recovered from this shift in the relative resistance change with only about 5 $\frac{\Omega }{\Omega }$ difference compared to the original relaxed state, which can be attributed to the same relative resistance change observed over the first strain cycle in the hysteresis analysis, after which the sensor behavior was stabilized. 

Environmental effects like response to temperature changes were not explicitly studied in this work but could be compensated using standard techniques like a compensating Wheatstone bridge [74,75]. 

### 3.7. Future Work

This research can be a foundation for studies focused on effects of environmental conditions, like temperature, humidity, and adhesion of the rGO to the PDMS, on the response of the sensor and the stability of the sensor. 

## 4. Conclusions 

This paper presented an investigation into the unique properties exhibited by reduced graphene oxide (rGO) when used as a resistive strain sensor undergoing large strains. Experimental results were produced for the resistive response aligned with ($\hat{x}$), perpendicular to ($\hat{y}$), and at a 45° angle to the applied strain. These results showed a unique linear/nonlinear behavior that was correlated to mechanical and parallel electrical theory.

The electrical resistance in the direction of applied strain showed a linear relation to the strain in lower strains up to a certain strain (between 3% and 12%), matching the piezo-resistive theory common to solid conductors. When the strain exceeded that certain strain, the behavior changed, producing a nonlinear relation between strain and resistance in the same direction, matching percolation theory commonly used to model the electrical behavior of particle-based conductors. This change in behavior coincided with formation and opening of mechanical cracks observed. 

While the gauge factor is a single linear fit measure of performance, not entirely appropriate for this application, it was calculated as 91.50 $\;\frac{\Omega /\Omega }{m/m}$ across the entire range, which was significantly greater than that of common foil strain gauges (gauge factor about 2) while being less expensive and easy to be synthesized. 

In the $\hat{y}$-direction, the experimental data showed a decreasing resistive trend, which matched the trends produced by mathematical modeling. The value of this resistive changes was significantly smaller compared to those in the $\hat{x}$-direction, which were attributed to a combination of the change in length in the $\hat{y}$-direction being governed by the Poisson effect and the orientation of crack formation, dominantly in the $\hat{y}$-direction, perpendicular to applied strain and parallel to the conductive path being investigated.

In the *45°* direction, both the experimental data and mathematical modeling showed that the resistive response was affected by the resistive responses in the $\hat{x}$- and $\hat{y}$-directions, with the $\hat{x}$-direction being the prevailing one. 

The AFM images suggested that due to existence of residual micro-cracks in the beginning of cycles 2, 3, and 4 and the fact that these cracks remained in place for the following cycles, the electrical response of the sensor starting from second cycle follows a similar pattern, contributing to the relatively smaller variation between cycles.

## Data Availability

The raw data supporting the conclusions of this article will be made available by the authors on request.
